# Changes in protein expression due to metformin treatment and hyperinsulinemia in a human endometrial cancer cell line

**DOI:** 10.1371/journal.pone.0248103

**Published:** 2021-03-09

**Authors:** Carsten Lange, Amanda Machado Weber, Ronny Schmidt, Christoph Schroeder, Thomas Strowitzki, Ariane Germeyer

**Affiliations:** 1 Department of Gynecologic Endocrinology and Fertility Disorders, Women’s Hospital, Ruprecht-Karls University of Heidelberg, Heidelberg, Germany; 2 Sciomics GmbH, Heidelberg, Germany; Michigan State University, UNITED STATES

## Abstract

The incidence of endometrial cancer (EC) has increased over the past years and mainly affects women above the age of 45 years. Metabolic diseases such as obesity and type II diabetes mellitus as well as associated conditions like polycystic ovary syndrome (PCOS), insulin resistance and hyperinsulinemia lead to elevated levels of circulating estrogens. Increased estrogen concentrations, in turn, further trigger the proliferation of endometrial cells and thus promote EC development and progression, especially in the absence of progesterone as seen in postmenopausal women. Elevated blood glucose levels in diabetic patients further contribute to the risk of EC development. Metformin is an insulin-sensitizing biguanide drug, commonly used in the treatment of type II diabetes mellitus, especially in obese patients. Besides its effects on glucose metabolism, metformin displayed anti-cancer effects in various cancer types, including EC. Direct anti-cancer effects of metformin target signaling pathways that are involved in cellular growth and proliferation, e.g. the AKT/PKB/mTOR pathway. Further proteins and pathways have been suggested as potential targets, but the underlying mechanism of action of metformin’s anti-cancer activity is still not completely understood. In the present study, the effects of metformin on protein expression were investigated in the human EC cell line HEC-1A using an affinity proteomic approach. Cells were treated with 0.5 mmol/L metformin over a period of 7 days and changes in the expression pattern of 1,300 different proteins were compared to the expression in untreated control cells as well as insulin-treated cells. Insulin treatment (100 ng/mL) was incorporated into the study in order to implement a model for insulin resistance and associated hyperinsulinemia, conditions that are often observed in obese and diabetic patients. Furthermore, the culture medium was supplemented with 10 nmol/L ß-estradiol (E2) during treatments to mimic increased estrogen levels, a common risk factor for EC development. Based on the most prominent and significant changes in expression, a set of 80 proteins was selected and subjected to a more detailed analysis. The data revealed that metformin and insulin targeted similar pathways in the present study and mostly acted on proteins related to proliferation, migration and tumor immune response. These pathways may be affected in a tumor-promoting as well as a tumor-suppressing way by either metformin treatment or insulin supplementation. The consequences for the cells resulting from the detected expression changes were discussed in detail for several proteins. The presented data helps identify potential targets affected by metformin treatment in EC and allows for a better understanding of the mechanism of action of the biguanide drug’s anti-cancer activity. However, further investigations are necessary to confirm the observations and conclusions drawn from the presented data after metformin administration, especially for proteins that were regulated in a favorable way, i.e. AKT3, CCND2, CD63, CD81, GFAP, IL5, IL17A, IRF4, PI3, and VTCN1. Further proteins might be of interest, where metformin counteracted unfavorable effects that have been induced by hyperinsulinemia.

## 1. Introduction

Endometrial cancer (EC) is the 6^th^ most common cancer in women and constitutes the leading malignancy of the female reproductive system in developed countries [1]. EC can be subdivided into an estrogen-dependent type I, and a less common (15–25%) but more aggressive and invasive, estrogen-independent type II [2–4]. The incidence of EC has increased over the past years and mainly affects postmenopausal women while it is uncommon to be diagnosed under the age of 45 years [5, 6]. The high incidence and mortality in developed countries could be related to a higher prevalence of common risk factors such as obesity and type II diabetes mellitus [1, 7, 8]. Obesity and obesity-associated diseases such as polycystic ovary syndrome (PCOS) lead to elevated levels of circulating estrogens that, in turn, increase proliferation of endometrial cells and promote EC development, especially in the absence of progesterone [9–11]. Elevated blood glucose levels of diabetic patients may further contribute to the risk of EC development via an increased glycolytic-lipogenic metabolism and the insulin-like growth factor (IGF) signaling pathway [12, 13].

Metformin is an insulin-sensitizing biguanide drug, commonly used in the treatment of type II diabetes mellitus [14], especially in obese patients [15]. Besides its effects on glucose metabolism, metformin displayed anti-cancer effects in various human cancer cell lines and diabetic patients with various cancer types [16–18], including EC [19–21]. The effects of metformin on malignant tissue may be direct or indirect. Indirect effects include a reduction in circulating glucose and insulin levels via a blockage of gluconeogenesis in the liver, which diminishes insulin resistance due to an inhibition of the IGF1 signaling pathway in tumor cells [22–24]. Direct anti-cancer effects of metformin target signaling pathways that are involved in cellular growth and proliferation, e.g. by activation of adenosine monophosphate-activated protein kinase (AMPK), a key regulator in energy homeostasis. Upon activation by liver kinase B1 (LKB1), AMPK induces various tumor suppressor genes, e.g. phosphatase and tensin homolog (PTEN), and thus downregulates growth-related pathways, especially mammalian target of rapamycin (mTOR) signaling [25]. Alterations in the phosphoinositide 3-kinase (PI3K)/protein kinase B (AKT/PKB)/mTOR pathway mediate EC growth and development and can be caused by a loss of PTEN or mutations of PI3K family members [26–30]. Additionally, metformin may act on growth and proliferation independent of AMPK, e.g. via downregulation of the proliferation marker Kiel-67 antigen (MKI67), via the transcription factor paired box gene 2 (PAX2), or via Ras-related guanosine triphosphate hydrolases (Rag GTPases) [25, 31–33]. Growing attention was also given to the inhibition of mitochondrial respiratory-chain complex 1 (NADH:ubiquinone oxidoreductase) by metformin in recent years. Like AMPK, the complex is involved in cellular energy homeostasis, but a direct involvement of AMPK in this mechanism is still a matter of debate [22, 34–36]. Further affected proteins and pathways like the inhibition of signal transducer and activator of transcription 3 (STAT3) [37], various routes of apoptosis induction [38], or regulation of microRNA (miRNA) [39] have been suggested as potential targets for the biguanide drug, but the underlying mechanism of action of metformin’s anti-cancer activity is still not completely understood.

In the present study, the effects of metformin on the human EC cell line HEC-1A were investigated using an affinity proteomic approach. HEC-1A cells were derived from a moderately differentiated grade 2 endometrial adenocarcinoma of a 71-year-old woman and are characterized by a poor expression of estrogen receptor α (ERα) [40, 41]. However, HEC-1A cells express ERβ and G protein-coupled estrogen receptor 1 (GPER) and therefore maintain a low estrogen sensitivity. Therefore, HEC-1A cells represent a postmenopausal model with low sensitivity for ß-estradiol (E2), which are able to form E2 from estrone (E1) to some extent [42]. Cells were treated long-term with metformin over a period of 7 days and changes in the expression pattern of 1,300 different proteins were compared to the expression in untreated control cells as well as insulin-treated cells. Insulin treatment was incorporated into the study in order to implement a model for insulin resistance and related hyperinsulinemia, conditions that are often observed in obese and prediabetic patients [43]. Although the HEC-1A cell line is characterized by low estrogen sensitivity, cells were additionally provided with E2 in order to mimic increased estrogen levels in the present study. Elevated estrogen levels represent a common risk factor for EC development and progression and are therefore essential for a realistic *in vitro* simulation of the environment in EC patients and women with increased risk for EC development [9–11]. It was shown that E2 stimulates cellular proliferation, migration and growth of HEC-1A cells independent of ERα via a GPER-mediated activation of diacylglycerol kinase α (DGKα) [44]. With the presented data, we contribute to a better understanding of the anti-cancer activity of metformin as well as its underlying mechanism of action in EC cells.

## 2. Methods

### 2.1. Cell culture and metformin treatment

The human EC cell line HEC-1A (moderately differentiated grade 2 adenocarcinoma; HTB112, ATCC, Manassas, VA, USA) [40, 41] was cultured in Eagle’s minimal essential medium (MEM, 5.5 mmol/L glucose, equivalent to 100 mg/dL; Sigma-Aldrich, Munich, Germany) supplemented with 10% (v/v) charcoal-stripped fetal bovine serum (FBS; Gibco, Waltham, MA, USA), 1% (v/v) non-essential amino acids (Sigma-Aldrich), 100 μg/mL streptomycin and 100 U/mL penicillin G (Gibco) at 37°C and 5% CO_2_ in a humidified atmosphere. Cells were subcultured by detachment with 0.25% (v/v) trypsin/ethylenediaminetetraacetic acid (EDTA; Gibco) once a week and the medium was changed every 2–3 days. Experiments were carried out in 25 cm^2^ cell culture flasks (Greiner, Kremsmünster, Austria) with a seeding density of 1.0 × 10^5^ cells in 5 mL medium in duplicates. After seeding, cells were allowed to attach and grow for 24 h before treatment.

Cells were treated with either 0.5 mmol/L metformin or 100 ng/mL insulin in culture medium supplemented with 10 nmol/L ß-estradiol (E2; all reagents purchased from Sigma-Aldrich) for 7 days with medium changes and renewed treatments every 2 days. Control cells were treated with substance-free medium supplemented with 10 nmol/L E2.

### 2.2. Cell lysis and protein extraction

Cells were detached from the substrate surface by incubation with trypsin after a treatment period of 7 days. The medium was removed and substituted by fresh substance-containing medium 2 h prior harvesting. Aliquots of 5.0 × 10^6^ cells were transferred to 1.5 mL tubes, washed with ice-cold phosphate-buffered saline (PBS; Sigma-Aldrich) and pellets were collected from three independent experiments and stored at -80°C until further analysis.

### 2.3. Proteomic analysis

Proteins were extracted with scioExtract buffer (Sciomics, Heidelberg, Germany) and total protein concentrations were measured with the bicinchoninic acid assay (BCA; Thermo Fisher Scientific, Waltham, MA, USA) according to the manufacturer’s protocol. The samples were labelled at an adjusted protein concentration with scioDye 1 and scioDye 2 (Sciomics) for 2 h. The reaction was stopped by an exchange of the buffer to PBS and proteins were analyzed in a dual-color approach using a reference-based design on nine scioDiscover antibody microarrays (Sciomics) targeting 1,300 different proteins with 1,830 antibodies in quadruplicates. Arrays were blocked with scioBlock (Sciomics) on a Hybstation 4800 (Tecan, Maennedorf, Switzerland) and afterwards incubated competitively using a dual-color approach. After incubation for 3 h, slides were washed with PBSTT, rinsed with 0.1% (v/v) PBS as well as water and subsequently dried with N_2_.

### 2.4. Data acquisition and bioinformatic analysis

Slide scanning was conducted using a PowerScanner (Tecan) with identical instrument laser power and adjusted photomultiplier tube (PMT) settings. Spot segmentation was performed with the GenePix Pro 6 software (Molecular Devices, San José, CA, USA). Acquired raw data were analyzed using the linear models for microarray data (LIMMA) package [45] of R-Bioconductor [46] after uploading the median signal intensities. For normalization, a specialized invariant Lowess method was applied [47]. For the sample analysis, a one-factorial linear model was fitted with LIMMA resulting in a two-sided t-test or F-test, based on moderated statistics. All presented *p* values were adjusted for multiple testing by controlling the false discovery rate according to Benjamini and Hochberg [48]. Differences in protein abundance between samples were presented as log_2_-fold changes (log_2_FC). Proteins were defined as differentially expressed, if |log_2_FC| were ≥ 0.5 or the adjusted *p* values were ≤ 0.05. Proteins were defined as significantly differential for |log_2_FC| ≥ 0.5 and a simultaneous adjusted *p* value ≤ 0.05. All differential proteins that met one or both criteria were graphically displayed in an area-proportional Venn diagram using the BioVenn application [49] and were subjected to STRING (search tool for the retrieval of interacting genes/proteins) analysis for the visualization of protein networks [50]. Protein clusters were identified for biological processes and molecular functions based on the gene ontology (GO) database [51, 52]. The database for annotation, visualization, and integrated discovery (DAVID) [53–55] was used for a more targeted pathway analysis (GO and Kyoto encyclopedia of genes and genomes (KEGG) pathway database [56]) that distinguished between proteins regulated by either metformin or insulin treatment. Pathways with protein counts ≥ 10% of the total number of analyzed proteins that simultaneously displayed *p* values ≤ 0.001 (from DAVID analysis) were presented.

## 3. Results

**Fig 1:**
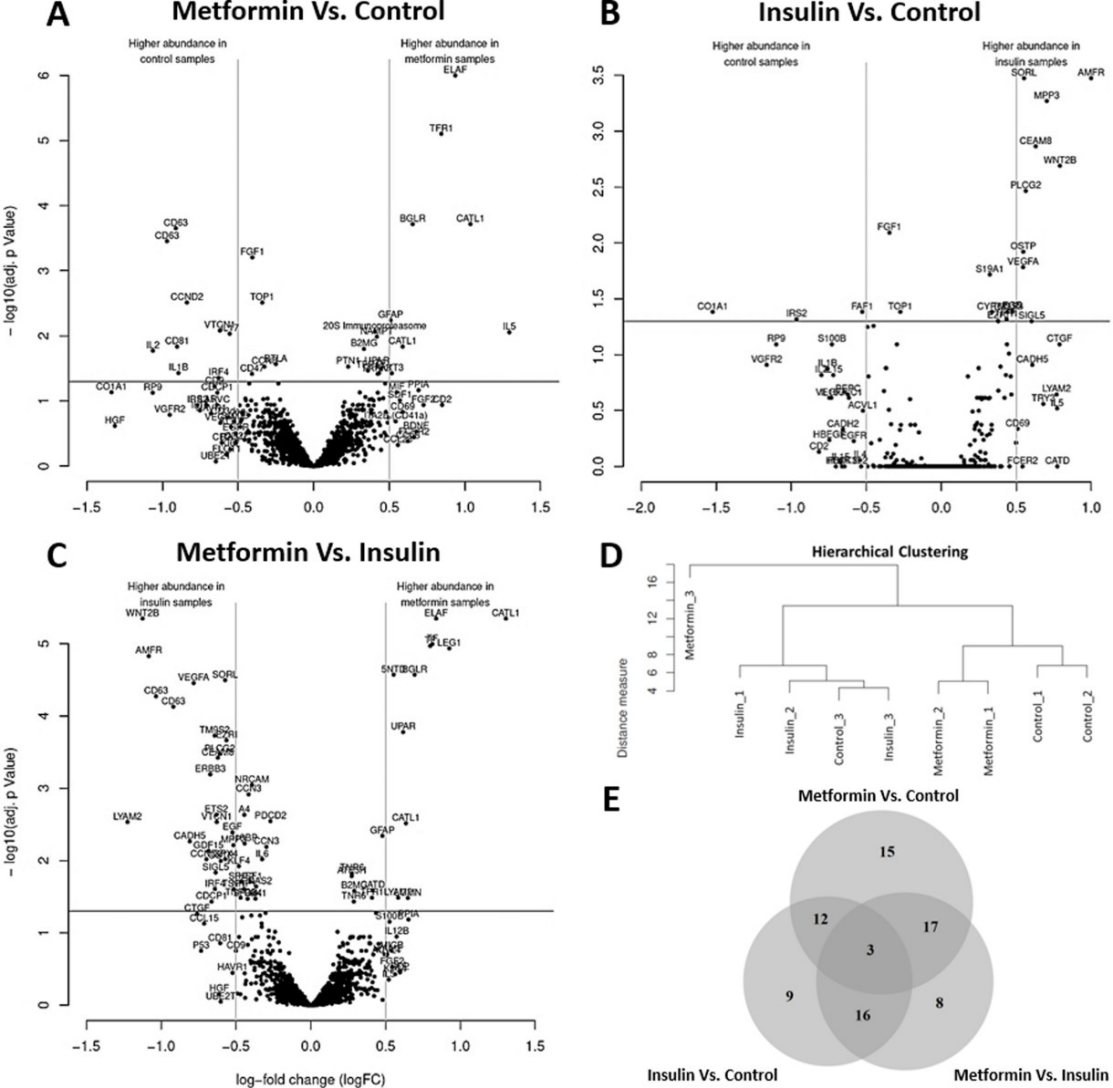
Proteomic analysis of endometrial cancer cells after treatment with metformin or insulin. Protein lysates of HEC-1A cells were collected 7 days after repeated administration of 0.5 mmol/L metformin or 100 ng/mL insulin and subjected to scioDiscover antibody microarrays targeting 1,300 different proteins. Untreated cells served as the reference control sample. Proteins were defined as significantly differential for |log_2_FC| ≥ 0.5 and an adjusted *p* value ≤ 0.05; n = 3. (**A**–**C**) Volcano plots for each treatment group display the distribution of proteins that were differently expressed after metformin (**A**) or insulin (**B**) administration compared to control samples or between metformin and insulin treatments (**C**). (**D**) Hierarchical clustering of samples based on differential protein data. One sample (Metformin_3) was identified as an outlier and removed from proteomic analysis. (**E**) Venn diagram of differentially expressed proteins after treatment with metformin or insulin (metformin/insulin vs. control) or between both treatment groups (metformin vs. insulin).

Hierarchical clustering of the samples was performed with the differential data set and a separation between the groups was observed except for one control sample (Control_3), which clustered with the insulin group (Fig 1D). Furthermore, one metformin sample (Metformin_3) was identified as an outlier and removed from the proteomic analysis.

Compared to untreated control cells, metformin-treated cells differentially expressed 47 proteins (with |log_2_FC| ≥ 0.5 and/or adjusted *p* value ≤ 0.05), of which 15 proteins were exclusively changed by the biguanide drug and not during hyperinsulinemia (Fig 1E and Table 1). A total of 15 out of 47 proteins met both of the above-mentioned criteria (log_2_FC| ≥ 0.5 and adjusted *p* value ≤ 0.05) and thus were significantly differential expressed in comparison to untreated control samples (Fig 1A, Table 1 and S1 Table). Additionally, differential changes in the expression of 12 out of 47 proteins overlapped with insulin-induced changes compared to control samples (Table 2) and 17 proteins were differentially expressed after metformin administration when compared to an hyperinsulinemic environment (CCND2, CD2, CD63, CD81, CDCP1, CTGF, F3, FGF2, GFAP, GUSB, HAVCR1, IRF4, PI3, PLAUR, PPIA, UBE2T, VTCN1). A total of 3 proteins were differentially expressed in all 3 considered groups (HGF, IL5, TFRC) (Fig 1E).

**Table 1:** Changes in protein expression after metformin treatment and insulin supplementation.

**proteins (15) affected by metformin treatment (|log**_**2**_**FC| ≥ 0.5), but not hyperinsulinemia (|log**_**2**_**FC| < 0.5)**	
upregulated:	AKT3, BDNF, CCL8, CCL28, CXCL12, ITGA2B, MIF
downregulated:	ARVCF, CD3E, CD9, CD22, IL17A, KLK3, MKI67, RAD51C
**proteins (15) with significantly differential expression due to metformin treatment (|log**_**2**_**FC| ≥ 0.5 and *p* ≤ 0.05)**	
upregulated:	AKT3, CTSL, GFAP, GUSB, IL5, PI3, TFRC
downregulated:	CCND2, CD63, CD81, IL1B, IL2, IL17A, IRF4, VTCN1
**proteins (9) affected by hyperinsulinemia (|log**_**2**_**FC| ≥ 0.5), but not metformin treatment (|log**_**2**_**FC| < 0.5)**	
upregulated:	CTSD, PRSS3, SPP1
downregulated:	CDH2, DKK3, FAF1, HBEGF, IGLC1, PGC
**proteins (11) with significantly differential expression due to hyperinsulinemia (|log**_**2**_**FC| ≥ 0.5 and *p* ≤ 0.05)**	
upregulated:	AMFR, CEACAM8, MPP3, PLCG2, SORL1, SPP1, VEGFA, WNT2B
downregulated:	COL1A1, FAF1, IRS2

Compared to untreated control cells, metformin-treated cells differentially expressed 47 proteins, of which 15 were exclusively changed by the biguanide drug (and not hyperinsulinemia). Insulin-treated cells differentially expressed 40 proteins, of which 9 were exclusively changed by the pancreatic hormone (and not metformin).

**Table 2:** Changes in protein expression between metformin- and insulin-treated cells.

**proteins (12) with differential expression compared to untreated controls (|log**_**2**_**FC| ≥ 0.5) that were regulated by metformin and hyperinsulinemia in the same way**	
upregulated:	CD69, FCER2
downregulated:	COL1A1, EGFR, FLOT1, IL1B, IL2, IL15, IRS2, KDR, RP9, S100B
**proteins (8) with significantly differential expression compared to untreated controls (|log**_**2**_**FC| ≥ 0.5 and *p* ≤ 0.05) that were significantly higher expressed after metformin treatment compared to hyperinsulinemia**	
CTSL, F3, GUSB, LGALS1, LGMN, NT5E, PI3, PLAUR	
**proteins (24) with significantly differential expression compared to untreated controls (|log**_**2**_**FC| ≥ 0.5 and *p* ≤ 0.05) that were significantly higher expressed during hyperinsulinemia compared to metformin treatment**	
AMFR, CCND2, CD63, CDCP1, CDH5, CEACAM8, EGF, ERBB3, ETS2, EZR, GDF15, GPX4, IRF4, MPP3, PLCG2, SELE, SIGLEC5, SORL1, SPP1, THBS1, TM9SF2, VEGFA, VTCN1, WNT2B	
**proteins (8) without differential expression compared to untreated controls (|log**_**2**_**FC| < 0.5), but with significantly differential expression between metformin treatment and hyperinsulinemia**	
ERBB3, ETS2, GDF15, GPX4, LGALS1, LGMN, NT5E, THBS1	

A total of 44 proteins were differentially expressed between metformin and insulin-treated groups, of which only 12 proteins were regulated in the same way. The remaining 32 proteins were regulated in different ways by metformin and insulin, of which 8 proteins were significantly higher expressed after metformin administration, whereas 24 proteins were significantly more prominent during hyperinsulinemia. A total of 8 proteins were differentially expressed between both treatments groups without notable changes compared to the respective untreated control samples.

Compared to untreated control cells, insulin-treated cells differentially expressed 40 proteins (with |log_2_FC| ≥ 0.5 and/or adjusted *p* value ≤ 0.05), of which 9 proteins were exclusively changed by the pancreatic hormone and not metformin (Fig 1E and Table 1). A total of 11 out of 40 proteins met both of the above-mentioned criteria (log_2_FC| ≥ 0.5 and adjusted *p* value ≤ 0.05) in the insulin resistance model and thus were significantly differential expressed in comparison to untreated control samples (Fig 1B, Table 1 and S1 Table). Additionally, 16 out of 40 proteins were differentially expressed due to insulin supplementation when compared to metformin-treated HEC-1A cells (ACVRL1, AMFR, CDH5, CEACAM8, CTGF, EGF, EZR, IL4, MPP3, PLCG2, SELE, SIGLEC5, SORL1, TM9SF2, VEGFA, WNT2B) (Fig 1E).

Furthermore, 44 proteins were differentially expressed between the metformin- and insulin-treated groups (with |log_2_FC| ≥ 0.5 and/or adjusted *p* value ≤ 0.05), of which only 12 proteins were regulated by metformin and insulin in the same way (Fig 1E and Table 2). A total of 32 out of 44 proteins met both of the criteria (|log_2_FC| ≥ 0.5 and adjusted *p* value ≤ 0.05), of which 8 proteins were significantly higher expressed after metformin treatment, whereas 24 proteins were significantly more prominent in insulin-treated cells (Fig 1C, Table 2 and S1 Table). A total of 8 out of 44 proteins were differentially expressed between both treatments groups without notable changes compared to the respective untreated control samples (Fig 1E and Table 2).

**Fig 2:**
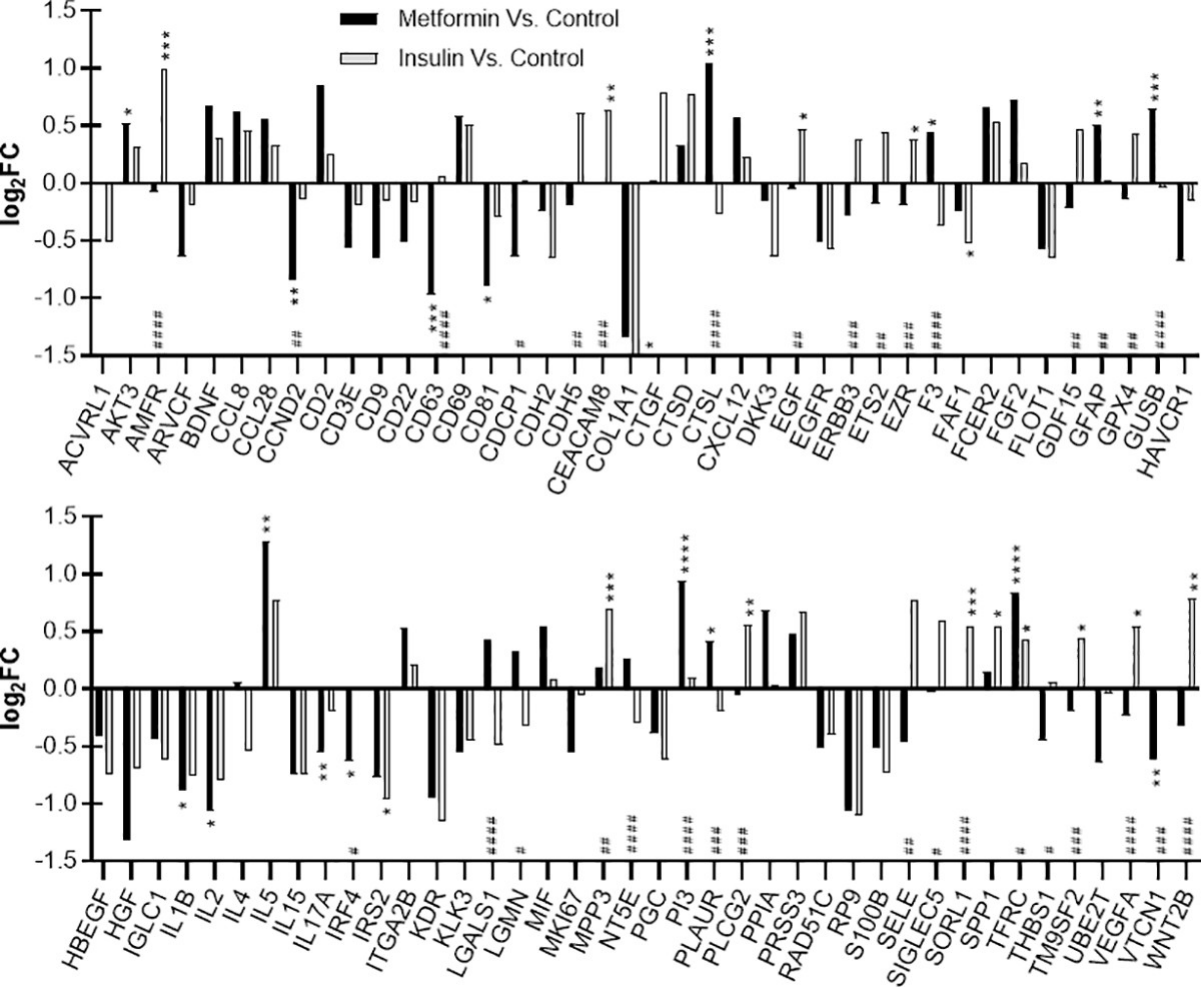
Expression of selected proteins in endometrial cancer cells after treatment with metformin or insulin. Differences in protein abundance between sample groups are presented as log_2_-fold changes (log_2_FC). Proteins were selected based on the following criteria: |log_2_FC| ≥ 0.5 and/or adjusted *p* value ≤ 0.05. Proteins were defined as significantly differential for |log_2_FC| > 0.5 and adjusted *p* values < 0.05; *p ≤ 0.05, **p ≤ 0.01, ***p ≤ 0.001, ****p ≤ 0.0001 (metformin/insulin vs. control); ^#^*p* ≤ 0.05, ^##^*p* ≤ 0.01, ^###^*p* ≤ 0.001, ^####^*p* ≤ 0.0001 (metformin vs. insulin).

A total of 80 proteins were differentially expressed (|log_2_FC| ≥ 0.5 and/or an adjusted *p* value ≤ 0.05) under one of the tested conditions (Fig 1E) and were plotted and further analyzed (Fig 2). A total of 17 proteins were significantly different from the control sample after metformin treatment (F3, PLAUR: |log_2_FC| < 0.5), whereas insulin significantly changed the expression of 15 proteins (EGF, EZR, TM9SF2: |log_2_FC| < 0.5). Of the 80 selected proteins, 34 proteins displayed a significantly different expression between both treatment groups (GFAP, TFRC: |log_2_FC| < 0.5). The results of the antibody microarray analysis have been confirmed for selected proteins, namely IL2 (ELISA) and COL1A1 (western blot analysis) (S1 Fig).

**Fig 3:**
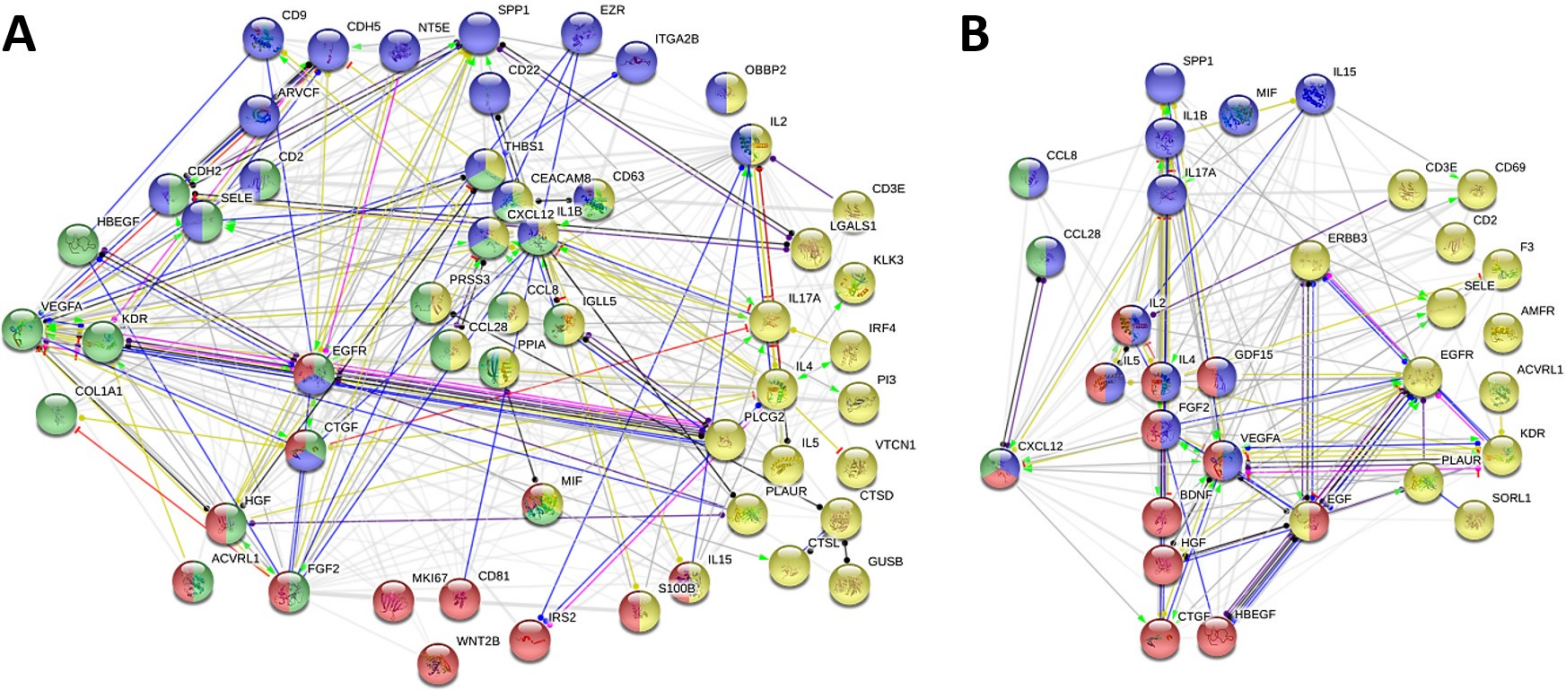
Network of proteins affected by metformin and insulin supplementation. Proteins that displayed a |log_2_FC| ≥ 0.5 and/or an adjusted *p* value ≤ 0.05 (total number: 80) were subjected to STRING analysis and protein clusters for biological processes (**A**, 53 proteins) or molecular functions (**B**, 31 proteins) were identified with the GO database. (**A**) Biological processes were divided into subsets related to cell population proliferation (red, 12 proteins), cell adhesion (blue, 20 proteins), cell migration (green, 23 proteins), and immune response (yellow, 29 proteins). A total of 27 proteins could not be assigned to any of the four biological processes (not shown). (**B**) Identified molecular functions included growth factor (red, 12 proteins), cytokine (blue, 14 proteins), chemokine (green, 3 proteins), and signal receptor activity (yellow, 13 proteins). A total of 50 proteins could not be assigned to any of the four molecular functions (not shown).

The 80 selected proteins were subjected to STRING analysis and protein networks were generated. Characteristic biological processes and molecular functions that were affected by the treatments were identified and proteins were assigned to clusters (Fig 3 and S1 Table). A total of 53 proteins were allocated to four different clusters of biological processes, of which 12 proteins were summarized in a subset related to cell population proliferation (e.g. CD81, MKI67, FGF2) (Fig 3A). Another two clusters contained 20 (e.g. CD63, CDH5, CEACAM8) and 23 proteins (e.g. CDH2, COL1A1, VEGFA) that were associated with cell adhesion and cell migration, respectively. A total of 29 proteins were related to immune response (e.g. CD3E, IL2, IL4). The remaining 27 proteins are not directly related to any of the above-mentioned biological processes.

Additionally, 31 proteins were assigned to four different clusters related to molecular functions, of which 12 proteins were summarized in a subset characterized by growth factor activity (e.g. CTGF, EGF, HGF) (Fig 3B). Another two clusters contained 14 (e.g. IL1B, IL2, IL17A) and 3 proteins (CCL8, CCL28, CXCL12) that were associated with cytokine and chemokine activity, respectively. A total of 13 proteins showed signal receptor activity (e.g. EGFR, ERBB3, KDR). The remaining 50 proteins could not be assigned to any of the above-mentioned molecular functions.

A more detailed pathway analysis of differentially expressed proteins distinguished between effects caused by either metformin or insulin compared to untreated HEC-1A control cells (metformin vs. control: 47 proteins; insulin vs. control: 40 proteins) and was carried out with the DAVID database (Tables 3 and 4). GO and KEGG annotations were considered relevant, if the following criteria were met: protein counts ≥ 10% (metformin vs. control: ≥ 5 counts; insulin vs. control: ≥ 4 counts) and *p* values ≤ 0.001 (from DAVID analysis).

**Table 3:** Pathway analysis for differentially expressed proteins that were affected by metformin compared to untreated HEC-1A control cells.

Identifier	Description	Counts	*p* value
**Cellular Component**			
GO:0005615	extracellular space	22	1.74×10^−12^
GO:0005576	extracellular region	20	3.47×10^−9^
GO:0070062	extracellular exosome	19	8.36×10^−5^
GO:0005887	integral component of plasma membrane	12	6.27×10^−4^
GO:0009897	external side of plasma membrane	10	2.60×10^−9^
GO:0009986	cell surface	8	3.87×10^−4^
GO:0005925	focal adhesion	7	4.26×10^−4^
**Molecular Function**			
GO:0005515	protein binding	37	2.20×10^−4^
GO:0005125	cytokine activity	7	7.82×10^−6^
GO:0008083	growth factor activity	6	7.63×10^−5^
GO:0005178	integrin binding	5	1.89×10^−4^
GO:0005088	Ras guanyl-nucleotide exchange factor activity	5	2.67×10^−4^
**Biological Process**			
GO:0008284	positive regulation of cell proliferation	10	3.29×10^−6^
GO:0006955	immune response	9	1.40×10^−5^
GO:0007155	cell adhesion	8	2.01×10^−4^
GO:0007166	cell surface receptor signaling pathway	7	8.53×10^−5^
GO:0006954	inflammatory response	7	4.92×10^−4^
GO:0030890	positive regulation of B cell proliferation	6	5.91×10^−8^
GO:0001934	positive regulation of protein phosphorylation	6	2.19×10^−5^
GO:0030335	positive regulation of cell migration	6	1.28×10^−4^
GO:0007267	cell-cell signaling	6	5.68×10^−4^
GO:0000165	MAPK cascade	6	6.53×10^−4^
GO:0042102	positive regulation of T cell proliferation	5	1.97×10^−5^
GO:0050731	positive regulation of peptidyl-tyrosine phosphorylation	5	6.76×10^−5^
GO:0045766	positive regulation of angiogenesis	5	2.50×10^−4^
GO:0006935	Chemotaxis	5	3.14×10^−4^
**KEGG Pathway**			
hsa04640	hematopoietic cell lineage	9	6.42×10^−9^
hsa04060	cytokine-cytokine receptor interaction	9	5.10×10^−6^
hsa04151	PI3K/AKT signaling pathway	9	1.45×10^−4^
hsa05205	proteoglycans in cancer	8	3.25×10^−5^
hsa04510	focal adhesion	7	3.38×10^−4^
hsa04672	intestinal immune network for IgA production	5	1.12×10^−4^
hsa05323	rheumatoid arthritis	5	9.14×10^−4^

The DAVID database was used for pathway analysis and GO and KEGG pathway identifiers are shown for protein counts ≥ 10% (metformin vs. control: ≥ 5 counts) of the total number of 47 analyzed proteins with *p* values ≤ 0.001 (from DAVID analysis).

**Table 4:** Pathway analysis for differentially expressed proteins that were affected by hyperinsulinemia compared to untreated HEC-1A control cells.

Identifier	Description	Counts	*p* value
**Cellular Component**			
GO:0005615	extracellular space	25	1.96×10^−18^
GO:0005576	extracellular region	20	5.88×10^−11^
GO:0070062	extracellular exosome	16	2.88×10^−4^
GO:0005887	integral component of plasma membrane	11	4.86×10^−4^
GO:0048471	perinuclear region of cytoplasm	9	3.44×10^−5^
GO:0005768	Endosome	8	3.63×10^−7^
GO:0009986	cell surface	7	8.27×10^−4^
GO:0009897	external side of plasma membrane	6	7.63×10^−5^
GO:0016323	basolateral plasma membrane	5	5.23×10^−4^
GO:0045121	membrane raft	5	8.66×10^−4^
**Molecular Function**			
GO:0005515	protein binding	30	3.77×10^−4^
GO:0008083	growth factor activity	8	4.35×10^−8^
GO:0005125	cytokine activity	7	1.77×10^−6^
GO:0005088	Ras guanyl-nucleotide exchange factor activity	6	4.28×10^−6^
GO:0005178	protein tyrosine kinase activity	5	1.78×10^−4^
**Biological Process**			
GO:0008284	positive regulation of cell proliferation	11	5.79×10^−8^
GO:0007165	signal transduction	11	1.90×10^−4^
GO:0030335	positive regulation of cell migration	7	3.29×10^−6^
GO:0000165	MAPK cascade	7	2.47×10^−5^
GO:0006955	immune response	7	3.35×10^−4^
GO:0007155	cell adhesion	7	5.30×10^−4^
GO:0030890	positive regulation of B cell proliferation	5	1.73×10^−6^
GO:0048015	phosphatidylinositol (PI)-mediated signaling	5	9.39×10^−5^
GO:0045766	positive regulation of angiogenesis	5	1.29×10^−4^
GO:0001934	positive regulation of protein phosphorylation	5	1.89×10^−4^
GO:0018108	peptidyl-tyrosine phosphorylation	5	3.84×10^−4^
**KEGG Pathway**			
hsa04151	PI3K/AKT signaling pathway	9	2.60×10^−5^
hsa05205	proteoglycans in cancer	8	6.50×10^−6^
hsa04510	focal adhesion	7	9.35×10^−5^
hsa04640	hematopoietic cell lineage	5	2.89×10^−4^
hsa04066	HIF1 signaling	5	4.21×10^−4^

The DAVID database was used for pathway analysis and GO and KEGG pathway identifiers are shown for protein counts ≥ 10% (insulin vs. control: ≥ 4 counts) of the total number of 40 analyzed proteins with *p* values ≤ 0.001 (from DAVID analysis).

Analysis of cellular components after metformin treatment revealed that most regulated proteins belonged to extracellular space/regions (19–22) and were mainly constituents of the plasma membrane (8–12), of which some were related to focal adhesion (7) (Table 3). Molecular functions of the affected proteins included protein binding (37), growth-related functions (6–7), and adhesion (5). The biological processes of the regulated proteins were related to cell proliferation (10), particularly of B and T cells (5–6), and with involvement of the MAPK cascade (6), cell adhesion, migration as well as angiogenesis (5–8), and immune (9) or inflammatory (7) responses. Furthermore, KEGG pathway analysis revealed a link to PI3K/AKT signaling (9), a pathway involved in quiescence, proliferation, and cancer. Cytokine-cytokine receptor interactions (9), focal adhesion (7), cancer-related components of the extracellular matrix, so called proteoglycans (8), and a link to hematopoiesis (9) were also identified as pathways that were targeted by metformin administration.

Likewise, analysis of cellular components after insulin treatment revealed that most regulated proteins belonged to extracellular space/regions (16–25) and were mainly constituents of the plasma membrane (5–11) or endosomes (8) (Table 4). Molecular functions of the affected proteins included protein binding (30), growth-related functions (7–8), and adhesion (5). The biological processes of the regulated proteins were related to cell proliferation (11), particularly of B cells (5), and with involvement of the MAPK cascade (7) and phosphatidylinositol (PI) signaling (5), cell adhesion, migration as well as angiogenesis (5–7), signal transduction (11), and immune responses (7). Furthermore, KEGG pathway analysis revealed a link to PI3K/AKT signaling (9), proteoglycans (8), focal adhesion (7), hematopoiesis (5), and hypoxia-inducible factor 1 (HIF1) signaling in a hyperinsulinemic environment.

## 4. Discussion

Besides its effects on glucose metabolism, the insulin-sensitizing drug metformin displayed anti-cancer effects in various cancer types [16–18], including EC [19–21]. Metabolic diseases like type II diabetes mellitus and obesity, together with increased unopposed estrogen levels, as seen in anovulatory women with PCOS as well as in women with prolonged reproductive phases including early menarche and late menopause, are known risk factors for EC development [9–11]. Metformin sensitizes the body to insulin effects and decreases hepatic gluconeogenesis *in vivo*, consequently leading to a reduction and thus to a normalization of blood glucose levels [57, 58]. In the present study, the direct effects of metformin on protein expression in the human EC cell line HEC-1A were investigated using an affinity proteomic approach. Insulin treatment was incorporated into the study in order to implement a model for insulin resistance, defined as normoglycemia reached by hyperinsulinemia, a condition often observed in obese and prediabetic patients [43]. Additionally, cells were treated in the presence of E2 to mimic increased estrogen levels, a common risk factor for EC development. Protein lysates of HEC-1A cells were collected 7 d after repeated administration of 0.5 mmol/L metformin or 100 ng/mL insulin and subjected to the scioDiscover antibody microarray targeting 1,300 different proteins. The aim of this study was to perform a proteomic screening after metformin as well as insulin treatment and to identify changes in protein expression that potentially contribute to the anti-cancer effects and the mechanism of action of the biguanide drug in EC.

Protein expression was compared to untreated controls and between metformin treatment and insulin supplementation (Fig 1). A total of 80 differentially expressed proteins were selected for further analysis based on most prominent changes (Fig 2). These proteins were subjected to STRING analysis for the generation of protein networks (Fig 3). Clusters of proteins were detected and proteins were assigned to four different biological processes including cell population proliferation, cell adhesion, cell migration and immune response (Fig 3A). Metformin has been found to act on these pathways in several studies before [24, 59–61]. Molecular functions of the affected proteins included growth factor, cytokine, chemokine, and signal receptor activities (Fig 3B).

A more detailed pathway analysis (GO and KEGG) of differentially expressed proteins discriminated between the effects caused by either metformin or insulin. The generated data from the DAVID database revealed that both metformin treatment and hyperinsulinemia targeted similar pathways (Tables 3 and 4). However, the regulated proteins varied among the treatment groups compared to untreated controls. Metformin and hyperinsulinemia changed the expression of extracellular proteins (e.g. metformin: CTSL, EGFR, IL5, KLK3; insulin: IL4, IL15, PGC, SORL1). Molecular functions of differentially regulated proteins were mainly related to binding, growth factor and immune-related cytokine activities (e.g. metformin: EGFR, IL1B, IL5, IL17A; insulin: EGFR, IL4, IL5, VEGFA). Affected biological processes included proliferation, adhesion, migration, angiogenesis and immune responses (e.g. metformin: CD3E, IL5, IL17A, TFRC; insulin: CEACAM8, CTGF, EGFR, IL4, IRS2). KEGG analysis identified adhesion, hematopoiesis and cancer-related proteoglycans as affected pathways (e.g. metformin: CD9, CTSL, IL5, HGF; insulin: EGF, EZR, PLCG2, TFRC). Furthermore, changes of proteins involved in PI3K/AKT signaling were detected (e.g. metformin: AKT3, CCND2, EGFR, FGF2; insulin: COL1A1, IL4, EGFR, VEGFA), a pathway that is a well-known target of metformin [16, 25, 59, 62, 63] and insulin [64–66].

Some proteins were regulated by metformin in a favorable way regarding tumor suppression and growth inhibition compared to untreated EC cells (Table 5), while they were not changed in regards to hyperinsulinemia. The following proteins were significantly downregulated by metformin, while no significant changes were detected in a hyperinsulinemic environment compared to the control: cyclin D2 (CCND2), cluster of differentiation 63 (CD63), CD81, interleukin-17A (IL17A), interferon regulatory factor 4 (IRF4), and V-set domain containing T cell activation inhibitor 1 (VTCN1, B7-H4). Downregulation of CCND2, a protein known to cause growth arrest in the G_1_ cell cycle phase, and CD81, a protein relevant for membrane organization, protein trafficking, cellular fusion and cell-cell interactions, contributes to a decreased proliferation, tumor progression, migration, and invasion [67–70]. Downregulation of CD63, a tetraspanin involved in the regulation of membrane protein trafficking, leukocyte recruitment and adhesion, was found to reduce apoptosis inhibition as well as cell survival [71], and to suppress vascular endothelial growth factor (VEGF) signaling as well as related angiogenesis [72]. In addition, metformin reduced the expression of several proteins related to immune responses, including VTCN1, a B7 family member involved in immune regulation, as well as IRF4, a regulator of lymphocyte growth and differentiation, leading to decreased tumor cell proliferation and metastasis [73–75]. Furthermore, IL17A is involved in immune responses and inflammation [76], but also promotes migration and invasion in cancer cells [77]. Therefore, a metformin-induced downregulation of the cytokine has the potential to inhibit migration and invasion. Ambiguous results were obtained after metformin-induced downregulation of IL1B and IL2. IL1B not only activates antigen-presenting cells, thereby initiating an adaptive anti-tumor response, but also promotes tumor growth, metastasis as well as angiogenesis upon activation by tumor-infiltrating macrophages. Therefore, downregulation of IL1B may decrease carcinogenesis and metastasis, but may also be able to block adaptive anti-tumor responses. Hence, IL1B-blocking drugs such as anakinra and canakinumab are currently investigated as cancer therapeutics [78, 79]. The cytokine IL2 is important for growth of T cells and natural killer cells, despite its regulatory effect in cell cycle progression and tumor cell growth [80]. On the one hand, downregulation of IL2 has been associated with decreased proliferation and carcinogenesis. On the other hand, no survival benefits were observed after administration of IL2 in metastatic renal cancer and melanoma, although the cytokine had led to a regression of metastatic tumors [81]. Therefore, the consequences of metformin treatment and hyperinsulinemia for the cellular fate are difficult to assess for these two interleukins and need to be further investigated to allow for clear conclusions.

**Table 5:** Favorable and unfavorable changes in the expression of significantly differential expressed proteins after metformin treatment.

significantly differential expressed proteins after metformin administration		
favorable regulation:	AKT3, CCND2, CD63, CD81, GFAP, IL5, IL17A, IRF4, PI3, VTCN1	(IL1B, IL2)
unfavorable regulation:	CTSL, GUSB, TFRC	

A significant protein upregulation compared to the untreated control was induced by metformin, but not by hyperinsulinemia, for immune regulatory factors such as IL5 and peptidase inhibitor 3 (PI3, elafin (ELAF)). IL5 is involved in the regulation and recruiting of eosinophils, which are critical for the suppression of tumor metastasis [82, 83], while an upregulation of PI3 counterbalances the mitogenic effects of the PI3 substrate elastase from activated neutrophils in the tumor microenvironment, thus reducing proliferation, tumorigenesis and migration [84, 85] as well as increasing apoptosis in tumor cells [86]. In addition, the overexpression of glial fibrillary acidic protein (GFAP), a component of intermediate filaments and hence a part of the cytoskeleton, is believed to decrease cell proliferation and tumor growth [87]. Similarly, upregulation of AKT3, an isoform of AKT and a major mediator of the cell cycle-regulating PI3K/AKT/mTOR signaling pathway, may decrease the motility and migration potential of cells [88, 89]. All the changes discussed above may be involved in a reduction of tumor progression and metastasis when metformin treatment is applied.

Unfavorable effects of metformin treatment may involve the upregulation of beta-glucuronidase (GUSB, BGLR), cathepsin L (CTSL, CATL1), and transferrin receptor 1 (TFRC, TFR1) (Table 5). GUSB expression has been correlated with increased invasion of tumor cells [90, 91], however, it remains unclear, whether an increased expression of GUSB is directly involved in tumor progression or is just upregulated during the process and rather serves as a marker for invasiveness [90]. Upregulation of CTSL leads to an increased degradation of components of the extracellular matrix (ECM), namely collagen and elastin, resulting in enhanced invasion and metastasis [92, 93]. TFRC is involved in the uptake of the iron-transporting glycoprotein transferrin into cells and helps meet the high demands for iron in tumor cells [94]. Hyperinsulinemia, however, did not change GUSB, slightly decreased CTSL expression and increased TFRC levels, all of these changes being significantly different from the metformin effects.

The following proteins were changed in a favorable way regarding tumor suppression and inhibition of invasiveness particularly under the influence of insulin (Table 6) and to a lesser extent by metformin in comparison to untreated control cells: collagen type I alpha 1 chain (COL1A1), and insulin receptor substrate 2 (IRS2). COL1A1, the major component of collagen type I, which, in turn, constitutes the main structural ECM protein, promotes cell migration and metastasis [95, 96]. Thus, downregulation of the ECM component, as found to be induced by both insulin and metformin in the present study, is considered as a desired effect. IRS2, a cytoplasmic adaptor protein, mediates the effects of insulin as well as IGF1 and therefore plays a role in growth-promoting IGF1 receptor (IGF1R) signaling. Overexpression is associated with increased glucose metabolism and tumor invasion as well as decreased overall survival and disease progression in patients [97, 98]. Therefore, the downregulation of IRS2 by hyperinsulinemia lowered tumor invasion and metastasis in the present study, which has also been proven in an obese and diabetic mouse model [99, 100]. Ambiguous results were obtained for carcinoembryonic antigen-related cell adhesion molecule 8 (CEACAM8) that was expressed in higher amounts after insulin supplementation. CEACAM8, a cell adhesion molecule expressed on the surface of neutrophilic granulocytes, has been associated with poor prognosis and decreased survival in patients [101, 102]. However, when bound to CEACAM6, upregulation of the CEACAM6/8 complex has been related to inhibition of vascular invasion and cell proliferation [103]. Little is known about the role of CEACAM8 in cancer [102], making it hard to assess the consequences of an insulin-induced CEACAM8 upregulation.

**Table 6:** Favorable and unfavorable changes in the expression of significantly differential expressed proteins during hyperinsulinemia.

significantly differential expressed proteins after insulin supplementation		
favorable regulation:	COL1A1, IRS2	(CEACAM8)
unfavorable regulation:	AMFR, FAF1, MPP3, PLCG2, SORL1, SPP1, VEGFA, WNT2B	

Besides the above-mentioned helpful effects observed in a hyperinsulinemic environment, elevated insulin levels also promoted a series of unfavorable changes (Table 6) in EC cell protein expression, including the upregulation of autocrine motility factor receptor (AMFR), FAS-associated factor 1 (FAF1), membrane palmitoylated protein 3 (MPP3), enzyme 1-phosphatidylinositol-4,5-bisphosphate (PIP_2_) phosphodiesterase gamma-2 (PLCG2, phospholipase Cγ2), sortilin-related receptor 1 (SORL1), secreted phosphoprotein 1 (SPP1, osteopontin (OSTP)), VEGFA, and wingless-type MMTV integration site family member 2B (WNT2B). AMFR, a cell surface receptor for AMF, is involved in many processes including cell motility, and AMFR upregulation has been related to enhanced tumor invasion and migration [104, 105]. WNT2B is involved in different cellular processes, including tumorigenesis, and WNT2B overexpression has been correlated with cancer progression and metastasis [106–108]. Likewise, upregulation of MPP3, a membrane protein involved in cytoskeleton organization and regulation of cell proliferation, signal transduction, and intracellular tight junctions has been associated with enhanced cell migration and invasion [109]. PLCG2 catalyzes the hydrolysis of PIP_2_ to form diacylglycerol (DAG) and inositol 1,4,5-trisphosphate (IP_3_), being important 2^nd^ messenger molecules that activate protein kinase C (PKC). PKC, in turn, is involved in signal transduction related to proliferation, differentiation, growth, and motility, among others [110, 111]. Downregulation of PLCG2 led to decreased cellular viability and proliferation in an *in vitro* model [112], and therefore it is likely that a hyperinsulinemia-mediated PLCG2 upregulation promoted cell proliferation. Overexpression of SPP1, a protein involved in ECM adhesion during wound healing, is also associated with cancer progression, metastasis, and apoptosis inhibition [113, 114], just like an upregulation of the transmembrane sorting protein SORL1 that promotes cell proliferation in cancer cells [115]. VEGFA mediates angiogenesis and vasculogenesis by regulation of endothelial cells and acts as a pro-survival factor of endothelial cells during tumor angiogenesis [116]. VEGFA overexpression, as seen after insulin supplementation in the present study, increased angiogenesis [116], while downregulation of VEGFA was not only found to inhibit cell proliferation, migration as well as invasion, but also to promote apoptosis [117]. Another unfavorable effect of hyperinsulinemia was identified by a downregulation of FAF1 in the present study, which was found to increase tumorigenesis [118], as FAF1 plays a role in various biological processes including apoptosis [119]. As metformin reduces peripheral hyperinsulinemia *in vivo*, the drug might be able to counteract the observed negative insulin effects when administered to patients with high levels of the pancreatic hormone. In addition, metformin acted on the expression of the discussed proteins in the opposite, more favorable way in the present study.

Several proteins have been expressed significantly different during metformin treatment and hyperinsulinemia, of which AMFR, CCND2, CD63, CEACAM8, CTSL, GFAP, GUSB, IRF4, PI3, PLCG2, SORL1, TFRC, VEGFA, VTCN1, and WNT2B have already been discussed above. Further proteins, that did not show high changes compared to untreated control samples (|log_2_FC| < 0.5), but displayed highly significant differences (*p* ≤ 0.001) among both treatments will now be discussed in more detail (Table 7). Changes in protein expression by metformin treatment displayed advantages over a hyperinsulinemic environment for the following proteins: erythroblastic oncogene B2 receptor tyrosine kinase 3 (ERBB3, human epidermal growth factor receptor 3 (HER3)), ezrin (EZR), and transmembrane 9 superfamily member 2 (TM9SF2). ERBB3 is a member of the epidermal growth factor receptor (EGFR) family and is directly involved in the pro-proliferative PI3K signaling pathway [120]. EZR is responsible for the linkage of the plasma membrane to the actin cytoskeleton and plays a key role in adhesion and migration [121, 122]. Little is known about the functions of TM9SF2 to date, but it is proposed to act as a channel or transporter [123]. Overexpression of ERBB3, EZR, and TM9SF2 has been associated with cancer progression, increased proliferation and metastasis or poor survival [120–122, 124, 125]. All three proteins were slightly downregulated after metformin administration, but upregulated in a hyperinsulinemic environment, with significant changes between both treatments. Therefore, metformin treatment is likely to promote anti-proliferative effects by the regulation of these proteins.

**Table 7:** Favorable changes in the expression of significantly differential expressed proteins between metformin treatment and hyperinsulinemia.

significantly differential expressed proteins between metformin- and insulin-treated groups	
favorable metformin effects:	AMFR, CCND2, CD63, ERBB3, EZR, GFAP, IRF4, PI3, PLCG2, SORL1, VEGFA, VTCN1, SPP1, TM9SF2
favorable insulin effects:	CEACAM8, CTSL, F3, GUSB, LGALS1, NT5E, PLAUR, TFRC

In contrast, the following proteins were regulated in a more favorable way during hyperinsulinemia compared to a treatment with metformin: coagulation factor III (F3, tissue factor (TF)), galectin-1 (LGALS1, LEG1), 5’-nucleotidase ecto (NT5E, 5NTD), and plasminogen activator urokinase receptor (PLAUR, UPAR). Overexpression of F3, a protein that initiates thrombin formation during hemostasis, effectively enhances angiogenesis as well as coagulation-associated metastasis [126]. LGALS1 covers various biological functions depending on its cellular location, e.g. when expressed intracellularly, LGALS1 contributes to tumor progression via immune suppression, angiogenesis and metastasis [127, 128]. NT5E is involved in cellular interaction with ECM proteins such as laminin and fibronectin and NT5E overexpression is known to support tumor proliferation, migration, angiogenesis, and immune escape [129]. The membrane-bound receptor PLAUR activates a cascade of extracellular proteinases with functions in tissue remodeling upon binding of its ligand, urokinase-type plasminogen activator (UPA), and upregulation of PLAUR promotes survival, migration and metastasis of tumor cells *in vitro* [130]. In the present study, the expression of all four discussed proteins was increased after metformin administration and decreased during hyperinsulinemia compared to untreated controls, with significant changes between both treatments. In these cases, metformin treatment is likely to suppress anti-proliferative effects and to favor tumor progression by the regulation of these proteins.

## 5. Conclusion

The proteomic analysis of changes in the expression of 1,300 different proteins in the human EC cell line HEC-1A revealed that both metformin treatment and insulin supplementation led to anti-tumor as well as some tumor-promoting effects. A set of 80 proteins were selected for a more detailed analysis based on most prominent changes. Metformin and insulin targeted similar pathways and mostly acted on proteins related to proliferation, migration, and changes in the tumor microenvironment, especially cellular immune response. The presented data helps identify proteins affected by metformin treatment as well as insulin supplementation in EC and allows for a better understanding of the mechanism of action of the anti-cancer properties of the biguanide drug, that are still not fully understood. However, further investigations are necessary for selected proteins to confirm the observations and conclusions drawn from the presented data after metformin administration, especially for proteins that were regulated in a favorable way, i.e. AKT3, CCND2, CD63, CD81, GFAP, IL5, IL17A, IRF4, PI3, and VTCN1. In the context of a hyperinsulinemic environment, as seen in obese women or PCOS patients, further proteins might be of interest, i.e. AMFR, CCND2, CD63, ERBB3, EZR, GFAP, IRF4, PI3, PLCG2, SORL1, VEGFA, VTCN1, SPP1, and TM9SF2, because sensitization to insulin due to metformin administration might be able to counteract unfavorable effects on their expression profile that have been induced by hyperinsulinemia.

## Supporting information

S1 Table Expression of selected proteins in EC cells after treatment with metformin or insulin. (PDF)

S1 Fig Expression of IL2 and COL1A1 in endometrial cancer cells after treatment with metformin or insulin. HEC-1A cells were treated with 0.5 mmol/L metformin or 100 ng/mL insulin. (**A**) Expression of IL2 was analyzed with an ELISA kit (HS200; R&D Systems, Minneapolis, Minnesota, MN, USA) according to the manufacturer’s protocol. (**B**) Expression of COL1A1 was analyzed with western blot analysis (ab138492; Abcam, Cambridge, UK). Data are presented as expression levels relative to the expression in untreated reference cells (log_2_FC).
(TIF)

S1 Raw images (PDF)
