# Supplementary material for: Changes in protein expression due to metformin treatment and hyperinsulinemia in a human endometrial cancer cell line
Source: PLoS One. 2021 Mar 9;16(3):e0248103. doi: 10.1371/journal.pone.0248103 (PMC7943011; doi:10.1371/journal.pone.0248103)
Supplement: S1 Table — (PDF) [file pone.0248103.s001.pdf]

Table S1. Expression of selected proteins in EC cells after treatment with metformin or insulin. Differences in protein abundance between samples are presented as log_2-fold changes (log_2FC), when $|log_2FC| \geq 0.5$ and/or adjusted *p* value ≤ 0.05. Proteins (total number: 80) were subjected to STRING analysis and clusters related to biological processes and molecular functions were identified with the help of the GO database.

Protein	Metf. Vs. Control		Ins. Vs. Control		Metf. Vs. Ins.		Cluster
	log_2FC	adj. *p*	log_2FC	adj. *p*	log_2FC	adj. *p*	
ACVRL1	-0.02	0.9876	-0.52	0.3174	0.51	0.2051	CP,CM;SR
AKT3/PKBG	0.52	0.0376	0.32	0.5131	0.19	0.6023	-
AMFR	-0.08	0.8488	1.00	0.0003	-1.08	≤ 0.0001	SR
ARVCF	-0.64	0.1148	-0.20	0.9999	-0.44	0.3616	CA
BDNF	0.68	0.2946	0.40	0.9999	0.28	0.7803	GF
CCL8	0.62	0.4130	0.46	0.9999	0.17	0.9035	CM,IR;CK,CH
CCL28	0.56	0.4744	0.33	0.9999	0.23	0.8621	CM,IR;CK,CH
CCND2	-0.84	0.0031	-0.14	0.9999	-0.70	0.0096	-
CD2	0.85	0.1148	0.25	0.9999	0.60	0.3511	CA,CM;SR
CD3E/CD3deg	-0.57	0.1769	-0.20	0.9999	-0.37	0.4923	IR;SR
CD9	-0.66	0.0595	-0.16	0.9999	-0.50	0.1770	CA
CD22	-0.51	0.4226	-0.17	0.9999	-0.34	0.6969	CA
CD63	-0.97	0.0004	0.06	0.9999	-1.03	≤ 0.0001	CA,CM,IR
CD69	0.59	0.1474	0.51	0.4601	0.08	0.9184	SR
CD81	-0.90	0.0147	-0.30	0.9999	-0.61	0.1386	CP
CDCP1	-0.64	0.0748	0.03	0.9999	-0.66	0.0369	-
CDH2	-0.25	0.6853	-0.66	0.4652	0.41	0.5780	CA,CM
CDH5	-0.20	0.6043	0.61	0.1235	-0.81	0.0054	CA
CEACAM8	0.01	0.9889	0.63	0.0014	-0.62	0.0004	CA,CM,IR
COL1A1	-1.34	0.0734	-1.52	0.0414	0.19	0.8991	CM
CTGF	0.03	0.9773	0.79	0.0808	-0.76	0.0544	CP,CA,CM;GF
CTSD	0.33	0.8177	0.77	0.9999	-0.44	0.7916	IR
CTSL/CATL1	1.04	0.0002	-0.27	0.9999	1.30	≤ 0.0001	IR
CXCL12/SDF1	0.57	0.0975	0.23	0.9999	0.33	0.4140	CA,CM,IR;GF,CK,CH
DKK3	-0.16	0.9488	-0.64	0.9999	0.48	0.8428	-
EGF	-0.05	0.8770	0.47	0.0405	-0.52	0.0041	GF,SR
EGFR	-0.52	0.3132	-0.58	0.5920	0.07	0.9525	CP,CA,CM;SR
ERBB3/HER3	-0.29	0.2417	0.38	0.2098	-0.67	0.0006	SR
ETS2	-0.18	0.5079	0.45	0.0979	-0.63	0.0023	-
EZR	-0.19	0.3563	0.38	0.0498	-0.56	0.0002	CA
F3/TF	0.44	0.0324	-0.37	0.1320	0.81	≤ 0.0001	SR
FAF1	-0.25	0.3609	-0.53	0.0414	0.28	0.3687	-
FCER2	0.66	0.3596	0.54	0.9999	0.12	0.9283	-
FGF2	0.73	0.1148	0.18	0.9999	0.54	0.2958	CP,CM;GF,CK
FLOT1	-0.58	0.6692	-0.66	0.9999	0.09	0.9883	-
GDF15	-0.22	0.5152	0.47	0.2272	-0.68	0.0073	GF,CK
GFAP	0.51	0.0058	0.03	0.9999	0.48	0.0046	-
GPX4	-0.14	0.6443	0.43	0.1523	-0.57	0.0096	-
GUSB/BGLR	0.65	0.0002	-0.04	0.9999	0.69	≤ 0.0001	IR
HAVCR1	-0.68	0.1555	-0.16	0.9999	-0.52	0.3569	-
HBEGF	-0.41	0.5531	-0.75	0.5801	0.34	0.7064	CM;GF
HGF	-1.32	0.2412	-0.70	0.9999	-0.61	0.7014	CP,CM;GF
IGLC1/IGLL5	-0.44	0.3072	-0.62	0.2432	0.17	0.7969	CM,IR
IL1B	-0.89	0.0373	-0.76	0.1335	-0.14	0.8679	CA,CM,IR;CK
IL2	-1.06	0.0169	-0.80	0.1523	-0.26	0.7061	CA,IR;GF,CK
IL4	0.06	0.9572	-0.54	0.8781	0.60	0.3323	IR;GF,CK
IL5	1.29	0.0088	0.77	0.3026	0.52	0.4416	IR;GF,CK
IL15	-0.75	0.1397	-0.75	0.2432	-0.01	0.9992	CP,IR,CK
IL17A	-0.56	0.0093	-0.19	0.9999	-0.37	0.1135	IR;CK
IRF4	-0.63	0.0444	0.01	0.9999	-0.64	0.0247	IR
IRS2	-0.77	0.1148	-0.96	0.0481	0.20	0.8054	CP
ITGA2B	0.54	0.2053	0.22	0.9999	0.32	0.5814	CA
KDR/VGFR2	-0.95	0.1633	-1.16	0.1235	0.21	0.8635	CM;SR
KLK3	-0.55	0.2501	-0.45	0.9786	-0.09	0.9184	IR
LGALS1/LEG1	0.43	0.0938	-0.49	0.0566	0.93	≤ 0.0001	IR
LGMN	0.33	0.3632	-0.32	0.9999	0.65	0.0329	-
MIF	0.55	0.0739	0.09	0.9999	0.45	0.1420	CP,CM,IR;CK
MKI67	-0.55	0.5531	-0.06	0.9999	-0.49	0.6881	CP
MPP3	0.19	0.4452	0.70	0.0005	-0.52	0.0061	-
NT5E/5NTD	0.26	0.1148	-0.30	0.0808	0.55	≤ 0.0001	CA
PGC/PEPC	-0.39	0.3720	-0.62	0.2272	0.23	0.6969	-
PI3/ELAF	0.94	≤ 0.0001	0.10	0.9999	0.84	≤ 0.0001	IR
PLAUR/UPAR	0.42	0.0298	-0.20	0.9786	0.62	0.0002	IR;SR
PLCG2	-0.05	0.8839	0.56	0.0034	-0.61	0.0003	IR
PPIA	0.69	0.0683	0.04	0.9999	0.65	0.0653	CM,IR
PRSS3/TRY3	0.48	0.3383	0.68	0.2757	-0.20	0.7901	CM,IR
RAD51C	-0.52	0.4371	-0.40	0.9999	-0.12	0.9206	-
RP9	-1.06	0.0748	-1.10	0.0808	0.04	0.9913	-
S100B	-0.52	0.2053	-0.73	0.0808	0.53	0.0701	CP,IR
SELE/LYAM2	-0.46	0.3939	0.77	0.2272	-1.23	0.0029	CA,CM;SR
SIGLEC5/OBBP2	-0.03	0.9545	0.60	0.0502	-0.64	0.0146	CA,IR
SORL1	-0.02	0.9456	0.55	0.0003	-0.57	≤ 0.0001	SR
SPP1/OSTP	0.15	0.5531	0.55	0.0120	-0.40	0.0576	CA;CK
TFRC/TFR1	0.84	≤ 0.0001	0.43	0.0481	0.41	0.0326	-
THBS1/TSP1	-0.45	0.0919	0.06	0.9999	-0.51	0.0248	CA,CM,IR
TM9SF2	-0.20	0.3745	0.44	0.0414	-0.64	0.0002	-
UBE2T	-0.65	0.8465	-0.04	0.9999	-0.60	0.8928	-
VEGFA	-0.24	0.3356	0.55	0.0165	-0.78	≤ 0.0001	CM;GF,CK
VTCN1/B7-H4	-0.62	0.0083	0.01	0.9999	-0.63	0.0029	IR
WNT2B	-0.33	0.2508	0.79	0.0020	-1.12	≤ 0.0001	CP

biological processes – CP: cell population proliferation, CA: cell adhesion, CM: cell migration, IR: immune response;
molecular functions – GF: growth factor activity, CK: cytokine activity, CH: chemokine activity, SR: signal receptor activity
