# Supplementary figures and images for: Changes in protein expression due to metformin treatment and hyperinsulinemia in a human endometrial cancer cell line
Source: PLoS One. 2021 Mar 9;16(3):e0248103. doi: 10.1371/journal.pone.0248103 (PMC7943011; doi:10.1371/journal.pone.0248103)

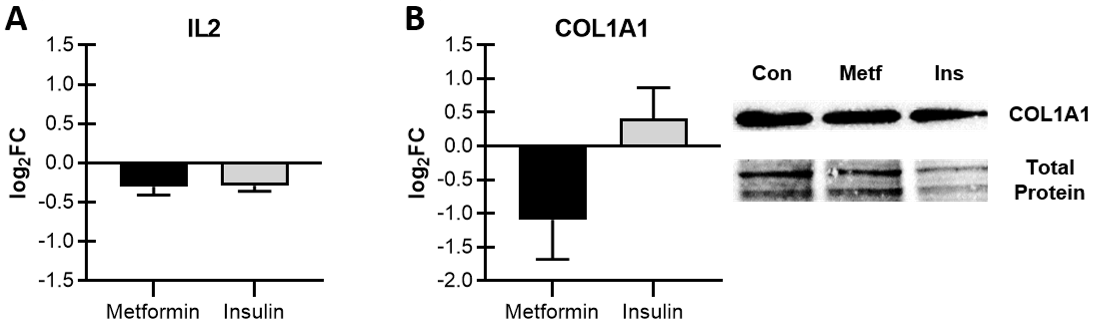

Supplement: S1 Fig — HEC-1A cells were treated with 0.5 mmol/L metformin or 100 ng/mL insulin. (A) Expression of IL2 was analyzed with an ELISA kit (HS200; R&D Systems, Minneapolis, Minnesota, MN, USA) according to the manufacturer’s protocol. (B) Expression of COL1A1 was analyzed with western blot analysis (ab138492; Abcam, Cambridge, UK). Data are presented as expression levels relative to the expression in untreated reference cells (log2FC). (TIF) [file pone.0248103.s002.tif]
