# Supplementary material for: Changes in protein expression due to metformin treatment and hyperinsulinemia in a human endometrial cancer cell line
Source: PLoS One. 2021 Mar 9;16(3):e0248103. doi: 10.1371/journal.pone.0248103 (PMC7943011; doi:10.1371/journal.pone.0248103)

SDS-PAGE gel (not shown in the manuscript)

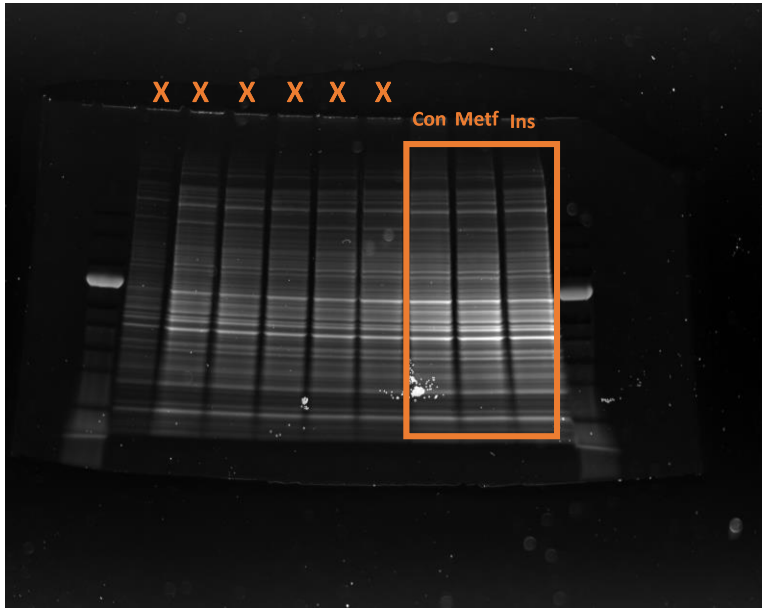

PVDF membrane after transfer (total protein; original; not shown in the manuscript)

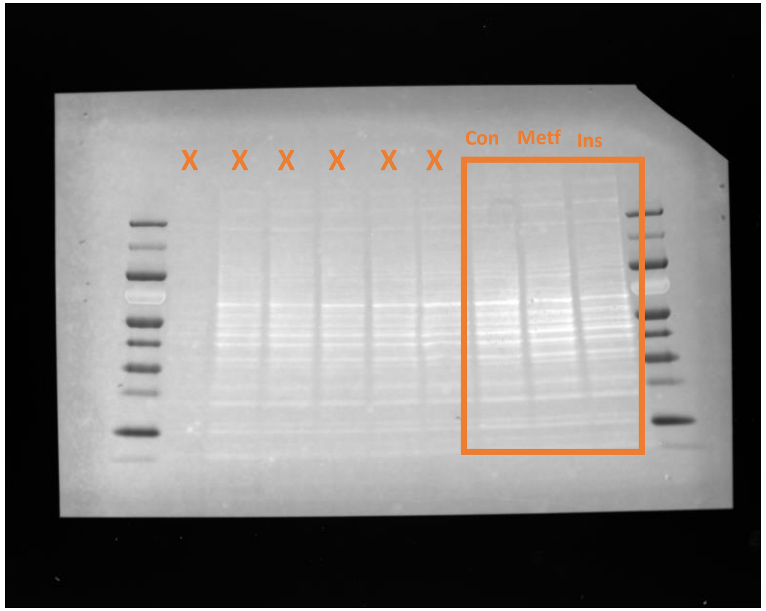

PVDF membrane after transfer (total protein; inverted as shown in the Supplementary Information)

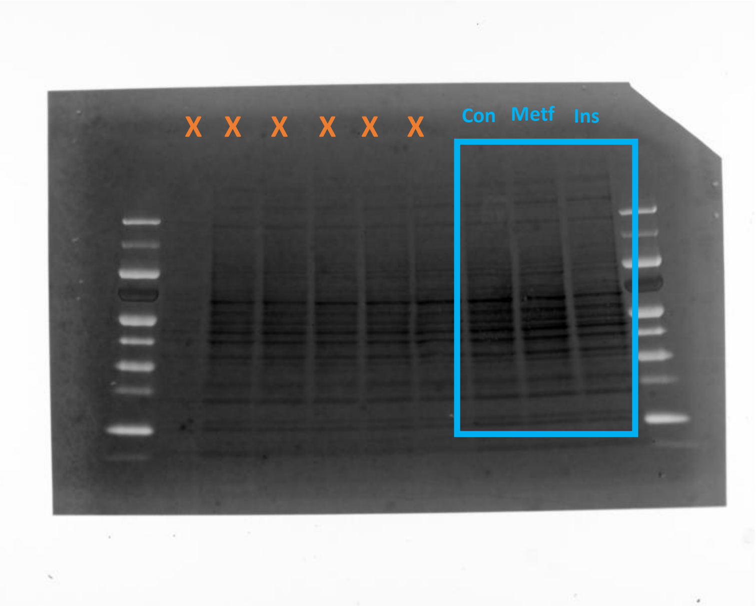

ECL signal for COL1A1 (original as shown in the Supplementary Information)

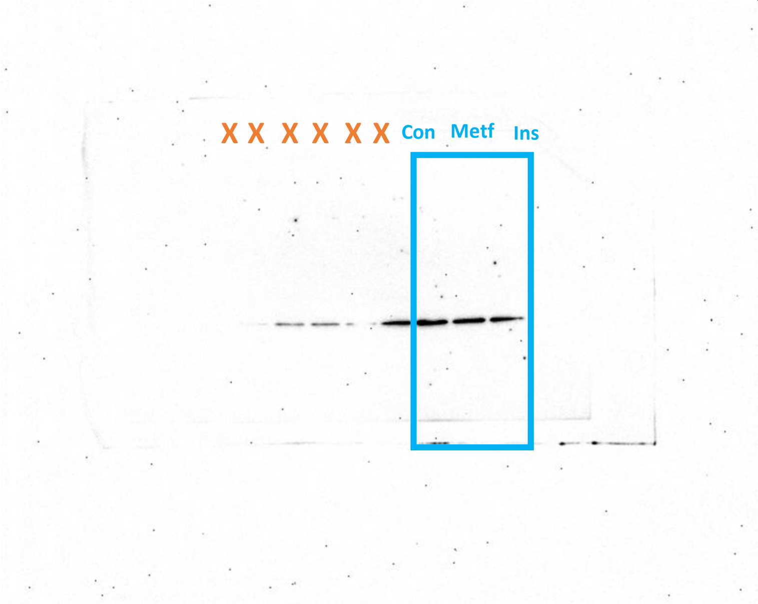

ECL signal for COL1A1 (marker overlay; not shown in the manuscript)

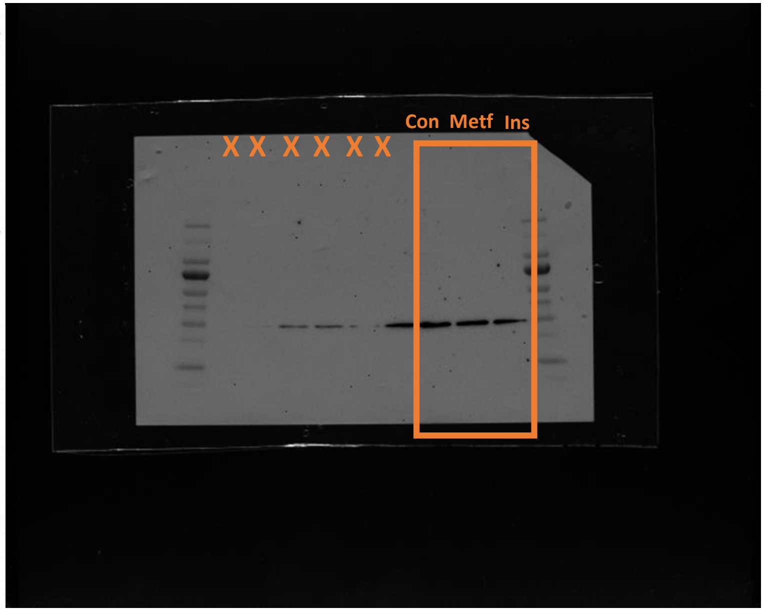

Supplement: S1 Raw images — (PDF) [file pone.0248103.s003.pdf]
